# Mechanical ventilation withdrawal as a palliative procedure in a Brazilian intensive care unit

**DOI:** 10.5935/0103-507X.20200090

**Published:** 2020

**Authors:** Fábio Holanda Lacerda, Pedro Garcia Checoli, Carla Marchini Dias da Silva, Carlos Eduardo Brandão, Daniel Neves Forte, Bruno Adler Maccagnan Pinheiro Besen

**Affiliations:** 1 Intensive Care Unit, Hospital da Luz - São Paulo (SP), Brazil.; 2 Intensive Care Unit, AC Camargo Cancer Center - São Paulo (SP), Brazil.; 3 Intensive Care Unit, Hospital OTOclínica - Fortaleza (CE), Brazil.; 4 Intensive Care Unit, Hospital Sírio-Libanês - São Paulo (SP), Brazil.; 5 Palliative Care Program, Hospital Sírio-Libanês - São Paulo (SP), Brazil.; 6 Intensive Care Unit, Medical Emergencies Discipline, Hospital das Clínicas, Faculdade de Medicina, Universidade de São Paulo - São Paulo, Brazil.

**Keywords:** Palliative care, Critical care, Respiration, artificial, Intensive care units, Cuidados paliativos, Cuidados críticos, Respiração artificial, Unidades de terapia intensiva

## Abstract

**Objective:**

To describe the characteristics and outcomes of patients undergoing mechanical ventilation withdrawal and to compare them to mechanically ventilated patients with limitations (withhold or withdrawal) of life-sustaining therapies but who did not undergo mechanical ventilation withdrawal.

**Methods:**

This was a retrospective cohort study from January 2014 to December 2018 of mechanically ventilated patients with any organ support limitation admitted to a single intensive care unit. We compared patients who underwent mechanical ventilation withdrawal and those who did not regarding intensive care unit and hospital mortality and length of stay in both an unadjusted analysis and a propensity score matched subsample. We also analyzed the time from mechanical ventilation withdrawal to death.

**Results:**

Out of 282 patients with life-sustaining therapy limitations, 31 (11%) underwent mechanical ventilation withdrawal. There was no baseline difference between groups. Intensive care unit and hospital mortality rates were 71% versus 57% and 93% versus 80%, respectively, among patients who underwent mechanical ventilation withdrawal and those who did not. The median intensive care unit length of stay was 7 versus 8 days (p = 0.6), and the hospital length of stay was 9 versus 15 days (p = 0.015). Hospital mortality was not significantly different (25/31; 81% versus 29/31; 93%; p = 0.26) after matching. The median time from mechanical ventilation withdrawal until death was 2 days [0 - 5], and 10/31 (32%) patients died within 24 hours after mechanical ventilation withdrawal.

**Conclusion:**

In this Brazilian report, mechanical ventilation withdrawal represented 11% of all patients with treatment limitations and was not associated with increased hospital mortality after propensity score matching on relevant covariates.

## Abstract (pt)

**Objetivo:**

Descrever as características e os desfechos de pacientes submetidos à retirada da ventilação mecânica e comparar a pacientes com ventilação mecânica e limitações de terapias de suporte à vida (limitar ou retirar), porém sem remoção da ventilação mecânica.

**Métodos:**

Este foi um estudo de coorte retrospectiva realizado entre janeiro de 2014 e dezembro de 2018 com pacientes em ventilação mecânica com alguma limitação de suporte artificial de vida admitidos a uma única unidade de terapia intensiva. Foram comparados os pacientes submetidos à retirada da ventilação mecânica e os que não passaram por esse procedimento com relação à mortalidade na unidade de terapia intensiva e ao tempo de permanência no hospital, em uma análise não ajustada e em uma amostra pareada por escore de propensão. Analisou-se também o tempo desde a retirada da ventilação mecânica até o óbito.

**Resultados:**

Dentre 282 pacientes com limitações a terapias de suporte à vida, 31 (11%) foram submetidos à retirada da ventilação mecânica. Não houve diferenças iniciais entre os grupos. As taxas de mortalidade na unidade de terapia intensiva e no hospital foram, respectivamente, de 71% *versus* 57% e 93% *versus* 80%, entre os pacientes submetidos à retirada da ventilação mecânica e os que não o foram. O tempo mediano de permanência na unidade de terapia intensiva foi de 7 *versus* 8 dias (p = 0,6), e o tempo de permanência no hospital foi de 9 *versus* 15 dias (p = 0,015). A mortalidade hospitalar não foi significantemente diferente (25/31; 81% *versus* 29/31; 93%; p = 0,26) após o pareamento. O tempo mediano desde a retirada da ventilação mecânica até o óbito foi de 2 dias [0 - 5] e 10/31 (32%) dos pacientes morreram dentro de 24 horas após a retirada dessa ventilação.

**Conclusão:**

Neste relato brasileiro, a retirada da ventilação mecânica representou 11% de todos os pacientes com limitação do tratamento e não se associou com aumento da mortalidade hospitalar após pareamento por escore de propensão das covariáveis relevantes.

## INTRODUCTION

During the hospitalization of a critically ill patient, clinical improvement may no longer be achieved, and invasive measures may conflict with patient and family values. The withdrawal of artificial life support can reduce the prolongation of unnecessary suffering and allow a dignified death.^(1,2)^ The opposite could be seen as dysthanasia and a potentially inappropriate use of resources.^(3,4)^

Mechanical ventilation withdrawal (MVW), also known as palliative extubation, is appropriate when mechanical ventilation (MV) is no longer in line with patient values according to prognosis and the likely outcomes. Although palliative extubation has been discussed for several years^(5,6)^ and even associated with relatives’ greater satisfaction with care,^(7)^there is still considerable worldwide variability in decisions to withhold/withdraw life-sustaining treatments (LSTs).^(8)^ Moreover, such decisions are less frequent in low- and middle-income countries.^(9)^

Although withholding LSTs is often preferred in Brazil,^(10)^ MVW has been increasingly studied and, as such, is recognized as an adequate procedure under some circumstances.^(11)^In Brazil, the decision to withdraw LSTs is associated with physicians’ education on end-of-life care and perception about futile procedures.^(10,12)^ However, MVW practices need further characterization.^(4,13)^Therefore, our objective was to characterize mechanically ventilated patients admitted to a single intensive care unit (ICU) with a decision to withhold or withdraw LSTs comparing those who underwent MVW to those who did not undergo MVW. We hypothesized that they would die sooner, but with similar hospital mortality.

## METHODS

This is a single-center, retrospective cohort study from January 2014 to December 2018. We included all mechanically ventilated patients with LST limitations (withholding or withdrawal). Life-sustaining treatments for the purposes of this definition were vasopressors, renal replacement therapy, MV or cardiopulmonary resuscitation during their ICU stay. We included only the last admission of each patient.

Consent to participate was waived by the Institutional Review Board (IRB) given the retrospective nature of the study (IRB approval: 1,700,252/CAAE: 58827116.0.0000.5533).

The ICU at *Hospital da Luz* is a mixed ICU consisting of 20 beds. Unit staffing included the following: one physician for every five beds during the morning and one physician for every 10 beds during the afternoon, night shift and weekend; one nurse for every seven beds during the day and for every 10 beds during the night shift; one nurse assistant for every two beds; one physical/respiratory therapist for every 10 beds, available 24 hours a day; and one psychologist from Monday to Friday for 6 hours a day. During the weekday mornings and afternoons, all physicians are trained intensivists with generalist-level knowledge on palliative care. During the weekends, one of the intensivists is responsible for the daily rounds. Three intensivists have postgraduate degrees in palliative care and act as consultants in difficult cases.

It is part of the ICU routine to address goals of care (GOCs) as soon as feasible after admitting patients with life-threatening conditions and whenever there is a futile or perceived potentially inappropriate ongoing therapy.^(14)^ In these cases, the physician schedules a meeting with the family - and, when possible, with the patient - to discuss GOCs and LST limitations. When MVW is decided as the best alternative, we use an institutional protocol as a guide for every palliative extubation, although we strongly advise against “one size fits all” and recommend an individual approach.

We retrieved variables of all patients during the study period from the prospectively collected ICU quality database (Epimed Monitor^®^).^(15)^ Measured baseline variables included age, sex, Simplified Acute Physiology Score (SAPS 3), Sequential Organ Failure Assessment (SOFA) score, premorbid functional status, comorbidities, Charlson comorbidity score, and the type of admission. We also retrieved the use of vasopressors, tracheostomy, MV, and renal replacement therapy at any time during ICU stay. Premorbid functional status was categorized in three ordinal categories as previously described: independent (Eastern Cooperative Oncology Group - ECOG^(16)^ 0 or 1), partially dependent (ECOG 2), and restricted/bedridden (ECOG > 2).

The primary outcome was hospital mortality. Secondary outcomes included ICU mortality, ICU and hospital length-of-stay (LOS), and time from MVW until death or hospital discharge.

The first author also reviewed the charts of patients who underwent MVW to discriminate the participants in the family meeting and to track the patient’s participation in the discussion of MVW. We looked for evidence of conflict - any disagreement between family members or between family members and healthcare workers - and any description of patient discomfort after extubation. This was considered if discomfort, breathing effort or stridor was described by any ICU staff in the patient’s chart.

We defined palliative extubation as MVW with an ad hoc discussion with relatives and other attending physicians about their extubation, the possibility of dying after the procedure and understanding that MVW would be performed to reduce the patient’s suffering, respecting the natural history of their disease. Some patients underwent extubation and had a *post hoc* decision not to intubate. These patients were not considered palliative extubation cases.

### Statistical analysis

Categorical variables are expressed as proportions. Continuous variables are expressed as the means and standard deviations or medians and percentiles [P25 - P75], as appropriate. We tested for normal distribution with the Shapiro-Wilk test and histograms. Categorical variables were compared with Fisher’s exact test. Student’s t-test or Wilcoxon’s rank-sum test were used to compare continuous variables. Among those who underwent MVW, we plotted Kaplan-Meier survival curves with MVW as the starting point.

First, we performed unadjusted comparisons between groups. Then, in an attempt to address confounding, we developed a propensity score based on a set of variables from a causal directed acyclic graph of the propensity to undergo MVW;^(17)^ i.e., characteristics that could influence the decision to proceed with MVW. The variables were age, pre-ICU hospital length of stay, SAPS 3, focal neurological deficit, coma, stroke sequelae, dementia, functional status, severe chronic obstructive disease (COPD), locoregional cancer, metastatic cancer, hematological cancer, and vasoactive drug use. We applied a 1:1 matching procedure with a caliper width of 0.1 and no replacement. The positivity assumption was evaluated with an overlap histogram. After matching, we compared variables using the paired T-test, the Wilcoxon *rank*-*sum test and* Fisher’s exact test, *as appropriate*. We used Stata SE 15.1 to conduct all analyses and the PSMATCH2 user-written command^(18)^ to perform the propensity score analysis. P-values < 0.05 were considered statistically significant, with no adjustments for multiple comparisons.

## RESULTS

**Figure 1:**
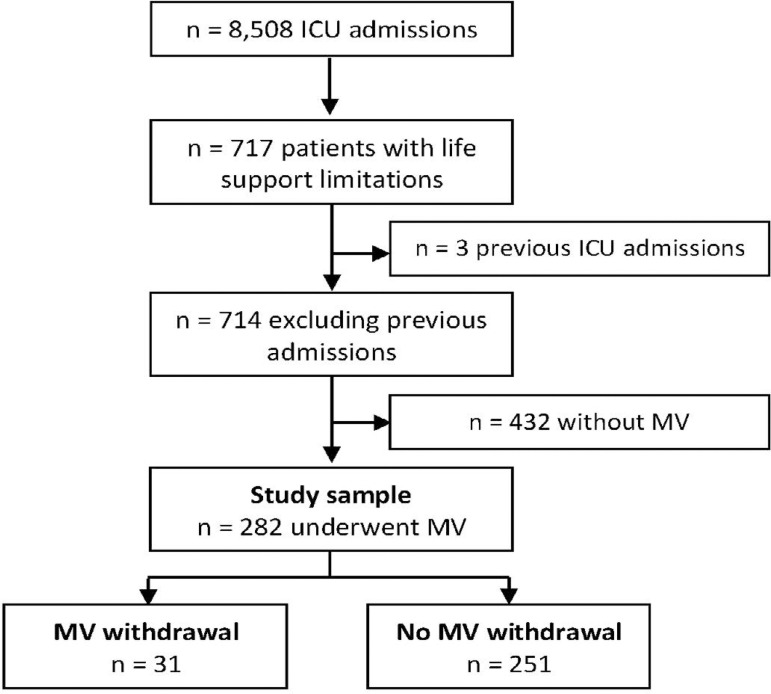
Patients’ selection. ICU - intensive care unit; MV - mechanical ventilation.

A total of 8,508 patients were admitted to the ICU during the study period. Of these, 714 (8.4%) had some limitation of artificial life support. Among these patients with LST limitation, we identified 282 (39.5%) undergoing MV and with some limitation of LST. A total of 31 (4.3%) underwent MVW (Figure 1). The sample characteristics are summarized in table 1. Patients were older age and had mostly medical conditions, with a high burden of comorbidities and an intermediate SOFA score. There was no baseline statistically significant difference between patients who did and did not undergo MVW.

**Table 1:** Baseline characteristics upon intensive care unit admission and outcomes of patients with life support limitations

	Without MVW n = 251	With MVW n = 31	p value
Age	75 [62 - 82]	74 [62 - 83]	0.91
Male	114 (46)	16 (53)	0.27
SOFA	5 [3 - 7]	6 [2 - 9]	0.36
SAPS 3	68 [60 - 80]	71 [60 - 80]	0.70
Coma	57 (23)	9 (29)	0.50
Focal deficit	11 (4)	1 (3)	> 0.99
Intracranial mass effect	1 (0.4)	0	> 0.99
Chronic kidney disease			
No dialysis	25 (10)	1 (3)	0.33
Dialysis	15 (6)	2 (6)	> 0.99
Heart failure			
NYHA 2-3	34 (13)	3 (9)	0.77
NYHA 4	6 (2)	0	> 0.99
Dementia	46 (19)	7 (23)	0.63
Stroke sequelae	24 (10)	3 (10)	> 0.99
Severe COPD	42 (17)	5 (16)	> 0.99
Cirrhosis Child A-B	3 (1)	0	> 0.99
Cirrhosis Child C	10 (4)	1 (3)	> 0.99
ICU readmission	38 (15)	2 (6)	0.27
Cancer			
Solid tumor locoregional	47 (19)	3 (9)	0.227
Solid tumor metastatic	36 (14)	5 (16)	0.79
Hematological malignancy	15 (6)	2 (6)	> 0.99
Status performance			0.36
Independent	143 (57)	19 (61)	0.70
Partial dependent	41 (16)	2 (6)	0.190
Restricted/bedridden	67 (27)	10 (32)	0.52
Charlson comorbidity index	3 [1 - 5]	2 [1 - 4]	0.209
Admission type			0.233
Medical	223 (89)	31 (100)	0.054
Emergency surgery	17 (7)	-	0.232
Elective surgery	11 (4)	-	0.61
Hospital LOS before ICU	0 [0 - 5]	0 [0 - 1]	0.111
Time to GOC discussion	5 [1 - 10]	3 [1 - 5]	0.040
Vasopressors	170 (67)	20 (64)	0.69
Noninvasive ventilation	33 (13)	2 (6)	0.39
Days on MV	4 [1 - 8]	4 [2 - 8]	0.49
RRT	30 (12)	0	0.057
Tracheostomy	27 (10)	0	0.054
ICU length of stay	8 [4 - 14]	7 [6 - 11]	0.64
Hospital length of stay	15 [8 - 25]	9 [7 - 17]	0.015
ICU mortality	144 (57)	22 (71)	0.177
Hospital mortality	203 (80)	29 (93.5)	0.131

MVW - mechanical ventilation withdrawal; SOFA - Sequential Organ Failure Assessment; SAPS - Simplified Acute Physiology Score; NYHA - New York Heart Association; COPD - chronic obstructive disease; ICU - intensive care unit; LOS - length of stay; GOC - goals of care; MV - mechanical ventilation; RRT - renal replacement therapy. Results expressed as percentile [P25 - P75] or n (%).

Most patients who underwent MVW died in the ICU (22/31; 71%) and two (6,5%) were discharged alive from the hospital. Mechanically ventilated patients who did not undergo MVW had an ICU mortality of 57% (144/251), and the hospital mortality was 80% (203/251) (Table 1). The median ICU LOS was the same in the patients who underwent MVW and those who did not (8 *versus* 7 days, p = 0.6), but median hospital LOS was different in the crude analysis (9 *versus* 15 days, p = 0.015).

Table 2 presents the data of the propensity-score-matched analysis (62 patients). Groups were mostly comparable. Although ICU mortality was different between groups (13/31; 42% *versus* 22/31; 71%; p = 0.040), hospital mortality was not significantly different (25/31; 81% *versus* 29/31; 93%; p = 0.26). The median ICU LOS was 5 *versus* 7 days, p = 0.126. The median hospital LOS was 15 *versus* 9 days, p = 0.153.

**Table 2:** Characteristics and outcomes of the propensity-score-matched cohort

	Without MVW n = 31	With MVW n = 31	p value
Age	77 [55 - 83]	74 [62 - 83]	0.74
SAPS 3	69 (15)	70 (15)	0.86
Coma	12 (39)	9 (29)	0.59
Stroke sequelae	1 (3)	1 (3)	> 0.99
Dementia	8 (26)	7 (22)	> 0.99
Severe COPD	3 (10)	5 (16)	0.71
Solid tumor, locoregional	0	3 (10)	0.238
Solid tumor, metastatic	1 (3)	2 (6)	> 0.99
Hematological malignancy	6 (19)	5 (16)	> 0.99
Status performance			0.53
Independent	15 (48)	19 (61)	
Partial dependent	4 (13)	2 (6)	
Restricted/bedridden	12 (39)	10 (32)	
Tracheostomy	2 (6)	0 (0)	0.49
Hospital LOS before ICU	0 [0 - 3]	0 [0 - 1]	0.30
RRT	2 (6)	0	0.49
Days on MV	4 [1 - 18]	4 [2 - 8]	0.71
Vasopressors	17 (55)	20 (64)	0.61
Hospital length of stay	15 [6 - 27]	9 [7 - 17]	0.153
ICU length of stay	5 [3 - 14]	7 [6 - 11]	0.126
ICU mortality	13 (42)	22 (71)	0.040
Hospital mortality	25 (81)	29 (93)	0.26
Time from GOC discussion to MVW	-	48 [24 - 120]	-
Time from MVW to death	-	2 [1 - 5]	-
Time from MVW to death (< 24 hours)*	-	16.5 [9 - 24]	-

MVW - mechanical ventilation withdrawal; SAPS- Simplified Acute Physiology Score; COPD - chronic obstructive disease; LOS - length of stay; ICU - intensive care unit; RRT- renal replacement therapy; MV - mechanical ventilation; GOC - goals of care.

* For patients who died within 24 hours of mechanical ventilation withdrawal. Results expressed as percentile [P25 - P75] or n (%).

**Figure 2:**
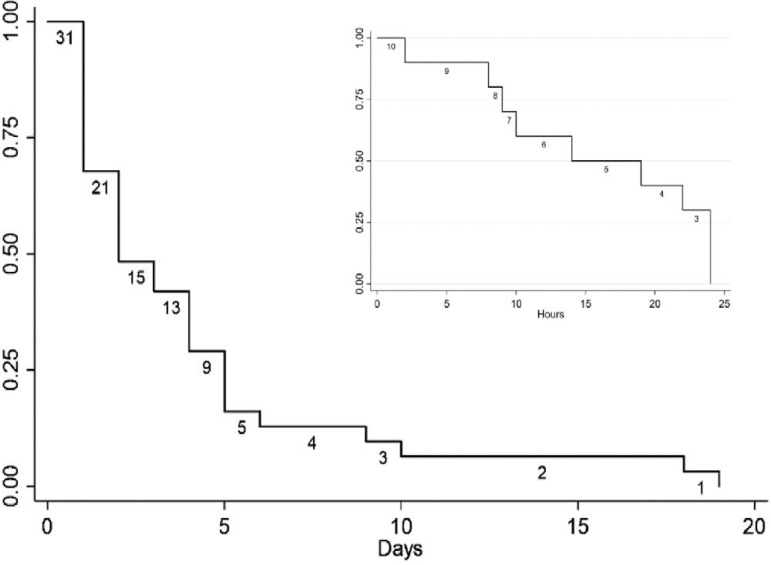
Survival after mechanical ventilation withdrawal. Kaplan-Meier plot showing the survival time after mechanical ventilation withdrawal. Twenty-one patients died after 24 hours with a median time of 2 days. Among those patients who died within 24 hours, the median time was 16.5 hours.

The family members present at family meetings where GOCs were discussed were mainly descendants (24/31, 77%). The median time from the definition of GOC to palliative extubation was 48 hours. Most patients died after 24 hours of MVW (21/31 patients), among whom the time until death ranged from 1 to 19 days (Figure 2). Those who died within 24 hours had a time until death that ranged from 2 to 24 hours.

There were no records of conflicts with family members or healthcare workers about MVW in the patients’ charts. Three patients (10%) could participate in discussions about palliative extubation: two while under MV and with controlled symptoms, and one discussed a time-limited trial before intubation and subsequently underwent MVW. Only three patients (10%) had stridor after extubation, but the discomfort could be quickly controlled.

## DISCUSSION

In this retrospective cohort study, 8.4% (717/8,508) of patients admitted to the ICU had a decision to withhold or withdraw LST. Out of these, 282 (39.5%) were under MV, of whom 31 (11%) underwent MVW. Despite no major differences in baseline characteristics, patients who underwent MVW had higher ICU mortality but the same hospital mortality, with a similar ICU LOS but a reduced hospital LOS. These results were similar in the propensity-score-matched analysis, although hospital LOS lost statistical significance, probably because of the low power of our sample. Furthermore, there was no evidence of either conflict during the process of MVW or difficult-to-treat discomfort after extubation. These results are encouraging and indeed show that MVW is acceptable for some families in Brazil, despite legal concerns among physicians.^(19)^

Our overall hospital mortality among extubated patients was no different from that reported in the literature,^(20)^ and the mortality of patients not undergoing palliative extubation was high (80%), although quite similar to that reported in a recent multicenter multinational cohort (69%).^(9)^ Interestingly, our time to death after MVW was not the same as that reported in other countries.^(21-24)^ Only 32% died within 24 hours, in contrast to 90% in the USA^(23)^ (possibly owing to a different case mix of palliative extubation patients), where the withdrawal of life support in the anticipation of death for patients with refractory shock or respiratory failure is more common.

Palliative care in Latin America is quite different from that in other regions.^(25)^ In Brazil, talking about death is unusual among many families. This cultural aspect brings some challenges when discussing the withdrawal of life support therapy with families. Sometimes, the family meeting may be even described as a painful moment. It takes time for the family to understand the prognosis of their loved one and that suffering may come from artificial life support. The best way to talk about this is by being honest, direct, and realistic^(26)^ and making sure that the family understands that we will continue taking care of their loved ones. As such, we must be aware of patient and family needs and avoid a damage to rapport that could hamper further attempts of MVW and end-of-life discussions.

In our ICU, if, after MVW, we realize that patients or family members need different care - i.e., continuous opioids or assuaging any insecurity over discharge - we avoid ICU discharge until the family is confident and the symptoms are easy to manage in the ward. Although it is speculative, we believe it has an explanation for the high mortality in the ICU with a long time for death after MVW. May be that the intensivists decided to continue symptom control and family care while the patient in the ICU due to the absence of a palliative care team in the ward. Only if optimal symptom control is achieved and there are no signs of imminent death would the patient be discharged to the wards with explicit orders of not being reintubated.

Our study has limitations: we cannot assume that these results are generalizable to every Brazilian ICU; the retrospective nature of this study precluded us from obtaining detailed information about the decision-making process to withdraw MV; propensity score matching, although useful to address confounders, may lead to a loss of data due to a reduction in sample size (we had low power to detect clinically important differences between groups, especially for hospital mortality, our primary outcome); and we did not follow-up with families, so we do not know the impact that discussing GOCs and deciding in favor of MVW could have on longer-term psychological outcomes.^(2,7)^

### Implications for practice

This report helps destigmatize the procedure of palliative extubation in Brazil and reinforces that the withdrawal of MV should not be viewed as dogma but as a feasible alternative in the situations where it can be applied. Nevertheless, we believe that other alternatives such as terminal weaning or the withdrawal of vasopressors may be considered alongside MVW according to the context and perceived risks of family members’ distress and psychological sequelae after their loved one’s death.

## CONCLUSION

In this Brazilian report, mechanical ventilation withdrawal was not associated with increased hospital mortality when compared to ventilated patients with life-sustaining therapy limitations who were not extubated, but hospital length-of-stay was shorter. Furthermore, mechanical ventilation withdrawal was acceptable to families and patients and was associated with hospital mortality rates similar to those in high-income countries. Further multicenter research is warranted in Brazil to evaluate intensive-care-unit-level characteristics that are associated with a higher propensity to include this procedure in daily routines.
